# Dying at “home” - a qualitative study of end-of-life care in rural Northern Norway from the perspective of health care professionals

**DOI:** 10.1186/s12913-023-10329-6

**Published:** 2023-12-05

**Authors:** Bente Ervik, Tom Dønnem, May-Lill Johansen

**Affiliations:** 1 Department of Oncology, University Hospital of North Norway, Tromsø, Norway; 2 Department of Clinical Medicine, UiT The Arctic University of Norway, Tromsø, Norway; 3 Research Unit for General Practice, Department of Community Medicine, UiT The Arctic University of Norway, Tromsø, N-9037 Norway

**Keywords:** Palliative care, End-of-life care, Home, Home place, Rural, Oncology nurse, Cancer nurse, Health care professionals, General practitioner (GP)

## Abstract

**Background:**

‘Most patients want to die at home’ is a familiar statement in palliative care. The rate of home deaths is therefore often used as a success criterion. However, providing palliative care and enabling patients to die at home in rural and remote areas may be challenging due to limited health care resources and geographical factors. In this study we explored health care professionals’ experiences and reflections on providing palliative care to patients at the end of life in rural Northern Norway.

**Methods:**

This is a qualitative focus group and interview study in rural Northern Norway including 52 health care professionals. Five uni-professional focus group discussions were followed by five interprofessional focus group discussions and six individual interviews. Transcripts were analysed thematically.

**Results:**

Health care professionals did their utmost to fulfil patients’ wishes to die at home. They described pros and cons of providing palliative care in rural communities, especially their dual roles as health care professionals and neighbours, friends or even relatives of patients. Continuity and carers’ important contributions were underlined. When home death was considered difficult or impossible, nurses expressed a pragmatic attitude, and the concept of home was extended to include ‘home place’ in the form of local health care facilities.

**Conclusions:**

Providing palliative care in patients’ homes is professionally and ethically challenging, and health care professionals’ dual roles in rural areas may lead to additional pressure. These factors need to be considered and addressed in discussions of the organization of care. Nurses’ pragmatic attitude when transfer to a local health care facility was necessary underlines the importance of building on local knowledge and collaboration. Systematic use of advance care planning may be one way of facilitating discussions between patients, family carers and health care professionals with the aim of achieving mutual understanding of what is feasible in a rural context.

**Supplementary Information:**

The online version contains supplementary material available at 10.1186/s12913-023-10329-6.

## Background

Studies of palliative care often include the statement ‘Most patients want to die at home’ [1–9], leading to an understanding of home death as the optimal goal for palliative patients [3, 10, 11]. Thus, a high proportion of home deaths in a country is often regarded as a sign of the success of the palliative care provided [2, 12]. Previous studies show that home is the preferred place of death, yet there is also great diversity of preferences [13–15]. A review by Gomes et al. [14] found that most people preferred to die at home, which was also found by Nilsson et al. among cancer patients [15]. Only about one-fifth of patients changed their preference for place of death as their illness progressed [13, 14].

However, although most patients prefer to die at home, this requires a certain level of competence and resources, and the patient, family carers and health care professionals (HCPs) must have a feeling of safety and security [16, 17].

Individual, structural and geographical factors all influence the place of death [7, 18]. A Norwegian study from 2017 [7] shows that being male and young increases the likelihood of dying at home. Further, the likelihood of dying at home increases when general practitioners (GPs) are part of the interdisciplinary team and provide home visits, and when patients receive intensive nursing home care at the end of life [7, 19].

In Norway, 14–15% died at home in 2015, and this percentage has remained stable in recent decades [8, 20], but with geographical variations. One of the main goals in the Norwegian Official Report on palliative care from 2017 [8] is that patients should have as much home time as possible at the end of life, and if possible, die at home if they want to. Kjeldstadli et al. [20] found that only 6.3% of deaths in Norway were potentially planned home deaths, and we still know little about palliative care and the possibility of home death from the perspective of HCPs in rural areas.

There are more home deaths in rural areas than in large towns and cities [21]. Between 1986 and 2015, hospital deaths decreased from 48 to 31%, while deaths in nursing homes increased from 27% to more than 48% [22]. The rise in the death rate in nursing homes can partly be explained by longer life expectancy, as many people spend their last months or years in nursing homes [7]. However, it is also a result of the government health policy of increasing the number of patients in community care, with a corresponding decrease in hospital stays [8, 23].

Studies comparing numbers of home deaths across European and other Western countries show significant differences, with Norway reporting a comparatively low rate [20]. However, a closer examination of the different studies reveals methodological challenges in comparing and interpreting the results, especially regarding the concept of home. In some countries home only includes private houses, while in other countries facilities such as nursing homes are defined as home [24]. In Norway, for statistical purposes, the place of death is divided into private homes, hospitals and ‘other health care facilities’, which includes all types of nursing and care homes [22]. Further, the organization of health care and access to health care resources varies within and between countries [18, 25].

Home has positive associations for most people [26], and primarily refers to a person’s place of residence. It is a place where one is surrounded by familiar objects that are meaningful in life. Home is also a household and a family setting, a place for relationships and togetherness, and probably a place where one has a specific role in the household, and thus a place of belonging. In the paper ‘Ideas of home in palliative care research: a concept analysis’, Tryselius et al. [11] also refer to the physical environment and significant others in their understanding of home. Martinsen [27] describes how various aspects of home have certain rhythms that are familiar to family members. Home is thus a place of inherent meaning and safety [11, 28].

Home is also a specific geographical place, comprising a unique, familiar environment. We all belong to a geographical place with a name that defines us, we are from somewhere, we are from home [29]. With reference to the Norwegian anthropologist Gullestad [30, 31], Broch [32] discusses the difference between the private and the public sphere in a local community. People from the same place have something in common, but their doorstep may be seen as protecting their privacy, and by crossing the doorstep one enters the person’s private sphere.

In an editorial, Bakitas and Dionne-Odom stated: ‘When it comes to death, there is no place like home … Or is there?’ [33]. The polarization between a home death as a good death and a death in hospital as a negative experience needs to be nuanced, and there is a need to reflect on different aspects of home and home death [24, 33]. In addition to previous research, our own experiences of cancer and palliative care from both primary and specialised health services have given us insights into the variation in how palliative care is provided in rural areas, and we still know little about HCPs’ own experiences.

### Aim

In this study we aimed to gain deeper insight into HCPs’ experiences and reflections on home and home death in rural Northern Norway.

## Methods

This paper is part of a larger study about HCPs’ experiences of providing palliative care in rural Northern Norway. The researchers’ different backgrounds added valuable insights and perspectives to the topic of this study. BE is a female oncology nurse, PhD and head of the Regional Advisory Unit for Palliative Care, TD is a male oncologist and professor, while MLJ is a female GP, PhD, and associate professor. We used an explorative and interpretative approach to investigate HCPs’ experiences. Ten focus group discussions and six individual interviews were conducted with a total of 52 HCPs; district nurses, oncology nurses, GPs, physiotherapists and occupational therapists in 2015–2016.

The study took place in Northern Norway, a region with a population of about 480 000, 87 municipalities and 11 hospitals of different sizes and specialities. Four of the municipalities had palliative care units with three or more beds, while 25 municipalities had one or two beds earmarked for palliative care in a nursing home.

### Data collection

First, we conducted five uni-professional focus group discussions. Three groups of nurses and two groups of GPs were interviewed separately to gain insight into their experiences without the presence of other professions. Each group consisted of 3–8 participants.

Nurses were recruited from participants on a palliative care course and two nursing network meetings. Participants were e-mailed by BE and 16 nurses from 14 different municipalities consented to participate and were interviewed in connection with the course and the network meetings. The first group of GPs was recruited by e-mail by MLJ and the interviews were conducted in connection with a meeting for GPs. The second GP group was recruited by snowballing from the first and this focus group discussion took place at a local hospital. Eight GPs from five municipalities participated in the two groups. In this first step, participants came from 19 different municipalities with a median population of about 3000 inhabitants.

To gain deeper insight into HCPs’ experiences with palliative care in their local communities, we purposively selected six rural municipalities by their different size, geography and organization of palliative care. We wanted a broad selection of experiences; hence, three municipalities had over 5000 inhabitants, while the other three had a population of 1200–2500. We also sought communities with long journeys to the nearest hospital, in some cases involving a ferry. Municipalities were also chosen based on our knowledge of how systematically palliative care had been developed, and whether they had oncology nurses.

Local oncology nurses or district nurses in these six municipalities were asked to find HCPs who usually collaborated in palliative care. In five municipalities, these professionals were invited to focus group discussions. All groups contained at least one district nurse, oncology nurse and GP. In four municipalities a physiotherapist also took part, while in one group both physiotherapists and occupational therapists participated. There were three to seven participants in each group. In one municipality, six individual interviews were conducted to gain insight into the experiences of each individual professional. A medical student (BB) conducted the individual interviews as part of her master’s degree, supervised by MLJ. Each individual interview lasted approximately 60 min. In this part of the study, the individual interviews and the focus group discussions were held in local health care centres or in nursing homes.

In the focus groups, we aimed for discussions among participants of different professions. We used a brief topic guide, published as an additional file in a previous paper [34], comprising four main topics: organization of palliative care, competence, interprofessional work and collaboration between primary and specialized care. Follow-up questions were asked to elicit reflections and narratives about home and home deaths, which are presented in this paper.

Each focus group discussion lasted around 90 min and was moderated by MLJ and BE. Both are experienced in qualitative research and focus group discussions. The participants gave their written informed consent, and all interviews were digitally recorded and transcribed verbatim. See Table 1 for an overview of participants.

**Table 1:** Participants in the study

Profession	N	Age range	Years of experience	Gender
District nurses	15	26–61	2–35	All women
Oncology nurses	15	35–60	9–37	All women
General practitioners	17	27–68	0–34	6 men, 11 women
Physiotherapists Occupational therapists	5	27–55	0–26	1 man, 4 women
Total	52			

## Analysis

We used a stepwise thematic analysis, including both semantic and latent content [35]. Initially, all interviews were carefully listened to by the first and third author in order to sort out the different voices and become familiar with the data. Following this, we independently coded the entire transcripts manually by taking notes and marking the text, looking for patterns, incidents and recurrent topics across the interviews related to the main research question of this paper. Transcripts containing the initial coding were discussed, and potential main themes and subthemes were then discussed between the researchers in a reiterative process. In this process we also used field notes. The different methods of data collection gave valuable insight into the study topic. The individual interviews and interprofessional FGDs supplemented and emphasized the information from the uni-professional FGDs. Bringing HCPs from different professions together to discuss their everyday collaboration also nuanced the information from the uni-professional FGDs.

The final themes were revised and refined until agreed upon. Themes were given names and illustrative quotes were selected to illuminate the content. Trustworthiness was assured by using an audit trail throughout the analysis process; to address credibility, we discussed our findings with colleagues with experience in palliative care. A COREQ checklist is available as an additional file.

## Results

HCPs did their utmost to fulfil patients’ wishes to die at home. They described the pros and cons of providing palliative care in small rural communities, and especially underlined continuity and the important contributions of informal carers. When a home death was considered difficult or impossible, they expressed a pragmatic attitude and being ‘at home’ was extended to include local health care facilities.

Four themes were found: (i) home as the private sphere, (ii) continuity in community care, (iii) family carers were indispensable, and (iv) ‘home place’: an extended understanding of home.

### Home as the private sphere

HCPs tried to arrange for home death for those patients wanting to die in their own home. Nurses described this as a moral obligation, they had a basic attitude of trying to fulfil patients’ wishes, and they often went the extra mile to achieve this goal.

*‘…when they know it’s about dying at home, well… I think maybe people get very involved in it and then they try… I think everyone tries as hard as they can, and maybe a bit more than that…’* (Muni 4, ON 2).

Home was primarily referred to as the physical house or flat, and nurses used phrases such as ‘*patients’ home ground’, ‘their (patients’) private arena’* and *‘a safe place with a special atmosphere’.* Some described home as a place of belonging where the patient had a certain role, a place where families could be together and patients could spend time in their favourite chair. Overall, they expressed a deep respect when entering a patient’s home; this called for humility and an obligation to do their very best. One nurse described how they entered the house step by step with dignity, another felt that entering a very personal place was ‘*almost like coming under the duvet’.*

Providing palliative care in the patient’s home was perceived as rewarding but also distressing. Many communities had a shortage of nurses, especially those with palliative care qualifications. Physiotherapists talked about valuable contributions: ‘*… for example, when the situation changed, so she got painful oedema… then you rang me and I sort of managed to squeeze it in, so I came…’* ( Muni 4, physio).

Further, patients often lived far from the centre of the village, where home care services were located. Thus, nurses often felt alone with a great deal of responsibility but without anyone to discuss their worries and problems with, as one district nurse narrated.

*‘…I was the only nurse in the municipality with… I got a patient* [from a hospital] *who was terminal and I was going to start using a palliative care kit and take all the decisions alone, observe everything all alone, observe everything all by myself. Should I start with morphine or should I give midazolam or… Should I be there all the time, how do we assess things? It’s often difficult in rural areas because you have to be…’* (FGD 2, DN).

### Continuity in community care

In some places, oncology nurses and GPs organized small teams around palliative patients and their families, which allowed them to use their complementary expertise in discussing expected or potential changes in the patient’s situation. Collaboration and continuity allowed HCPs to discuss the situation with the patients and their family when convenient, and make plans for the last months, weeks and days of life. HCPs described trying to anticipate a potential crisis to avoid the necessity of e.g. calling a helicopter to transport the patient, as suggested by this GP’s description.

*‘…there’s a big difference between patients you’ve followed over time and had contact with throughout. …you talk to them about the end and how they want it and what they think about… And then it’s much easier the day there’s a crisis, because then you’ve made a kind of plan for it… then at least the patient has a better chance of having a good end to life… if you’ve been able to be with him all the way.’* (FGD 2, GP 2).

Several GPs expressed their desire for continuity of care, preferably through a well-established relationship with the patient before a terminal illness. Living in small communities implied that HCPs often knew patients and their families. They could be neighbours, colleagues and even family members. Thus, many described identifying with patients and their family’s situation. Nurses often spent considerable time alone with the family in the final days of life, and therefore needed to find ways to deal with the emotional distress caused by the close involvement and their dual roles in the community. Many deaths in a short space of time put a great strain on nurses. GPs had one or two palliative patients each year on average, and spent less time in their homes than most nurses. GPs underlined that working closely with other HCPs and knowing each other well made it easier to help each other when necessary. On the other hand, one GP missed the opportunity to discuss patients with HCPs who were not involved in the community.

### Family carers were indispensable

Family carers were essential to allow patients to die at home, and nurses spent a great deal of time and energy informing and advising family carers to help them to feel secure in their caring role, and to be able to observe changes in the patient’s situation and know when to call for help. Despite close collaboration, some family carers did not understand that the patient was dying, and others were disappointed when HCPs could not be in their home and contribute to care as much as they needed and expected. In general, collaboration between patients, family carers and HCPs went well, and the last days and hours at home were calm. However, for some carers the situation became frightening and unmanageable. It was difficult to tell the patient that they could not cope with the situation at home. They therefore asked nurses to help them discuss the situation with the patient. These situations required a careful approach, and usually resulted in transfer to a local nursing home or a palliative unit. However, sometimes they found other solutions, as shown in the following description.

*‘…there was a man who wanted to be at home until the end. And he had his wife there and lots of children. But none of them could bear to be alone with… So then I was there from early evening until the next morning, until he died. And it was very strange… then I was in the sitting room with the patient, and then his wife and the others were sitting in the kitchen with a door between them… And they were terrified to come in.’* (Muni 5, ON 2).

### ‘Home place’: an extended understanding of home

Despite the best intentions, planning for home death was challenging. In addition to limited human resources, geographical factors played a part, most obviously in communities on islands without a ferry around the clock. Often patients lived alone in a remote location while their family members lived in the centre of the village or in more urban areas. Rapidly changing weather could prevent HCPs from reaching the house. Thus, in situations where home death was planned and prepared for, nurses often added ‘if possible’, and underlined the importance of not promising too much.

Transfer to a nursing home or hospital was sometimes due to a shortage of staff, but usually the situation was more complex, involving e.g. emergencies, infections and unmanageable symptoms. A transfer to a local facility was then seen as a natural development and a necessary next step. Nurses described the decisions on transfer in a pragmatic way, as a reality and not as ‘failure’ or a step backwards. Nursing homes were referred to as ‘home’, i.e. being in the home community in contrast to hospitals in more urban areas. ‘*It’s like coming home to (name of municipality) then. That’s fine, I can be discharged from hospital, but I’m going home to ‘my municipality’. …and then they often actually mean the nursing home.’* (Muni 4, physio).

As the initial plan for home death might turn out to be unfeasible, having a plan B was seen as important. The best scenario was therefore when the possibility of transfer to a nursing home was discussed before the situation became too burdensome.

*‘But it’s really important what you say about it not being a step backwards… For example, the day the patient is unable to move from his bed to the bathroom by himself. Then it’s time for the nursing home. I think you should agree about things like that in good time. Because then… and some other things too, you should agree on something or other that will mean it doesn’t seem like a big step backwards, like now we’ve reached…’* (FGD 1, DN).

Being close to family and friends and being in one’s local area were underlined as important for patients’ feeling of safety in a nursing home. The presence of palliative units or palliative beds was seen as positive since the HCPs had the necessary competence, including local cultural competence.

*‘…it’s not only when they’re at home that there must be proper competence, but also when they’re actually admitted to a ward for the last time… these are seriously ill patients, so you need a lot more than… I’d say you need staff who are specialized in palliative care.’* (FGD 1, GP 5).

## Discussion

In this study we aimed to gain deeper insight into HCPs’ reflections on home and home death in palliative care for patients in rural Northern Norway.

### The private sphere

Providing palliative care in the patient’s home can be understood as an exceptional situation for all involved. The expression *“coming under the duvet”* exemplifies the intimacy that can be experienced in a care context that requires emotional involvement and ethical reflection on maintaining the dignity of patients and their close ones [6, 36]. Further, the description of entering the home step by step is interesting, and may be seen as an understanding of entering a private sphere, which needs to be done with dignity. According to Brock and Gullestad [30, 31], the doorstep of a house marks a symbolic border between home as a personal space and the public sphere, and this distinction may be worthy of particular attention in rural areas where HCPs have different roles in the community [37].

### Continuity of care

Despite making great efforts to enable home death for those who wanted it, HCPs had to find a balance between patients’ and families’ wishes and needs and what was feasible in each particular situation. Nurses’ descriptions of feeling great responsibility for patients being at home support findings from a study in rural Canada, where Pesut et al. [38] found that close contact with patients and their families gave nurses a profound sense of responsibility for the quality of care for their patients. Nurses’ dual role in the community and intimate knowledge of patients and their families could enable better adjusted and more patient-centred care [3]. Similarly, being able to do good for a neighbour may be rewarding. However, the same close contact could increase emotional and moral distress, as in cases where nurses felt that they were expected to be more available for family carers [6]. Additionally, caring for many dying patients in a short space of time could imply a high burden of care.

GPs often mentioned the advantage of knowing their patients and being able to accompany them through their illness [39]. Some GPs emphasized that living in small communities where the HCPs knew each other well made it easier to collaborate and support each other in complex or challenging situations. Additionally, establishing small palliative teams around patients and their families may be one way to meet challenges in rural areas [34].

### Family carers

Extended family support has been shown to increase the likelihood of home death [15]; however, as shown in this study, providing end-of-life care at home may be exhausting for family carers. According to Ewing et al. [40], family carers often do not fully understand what it means for them to have a loved one dying at home. Our findings show that HCPs provided information, counselling and care for family carers, but for some carers this was not enough, and they wanted home care nurses to spend more time with them in their home. HCPs pointed out that they saw how hard it could be to care for loved ones at home. One example of this is the story about family members being in the kitchen while the nurse was with the dying patient in the sitting room.

According to Gomes et al. [41], the tendency towards dying at home increases when patients have discussed the situation with their family and the family is aware of the incurability of the disease. In recent years, systematic written care plans for palliative patients have been prioritized in Norway, thus many primary care services have implemented advance care planning in their daily work. Discussions on advance care planning enable patients and their close ones to talk about wishes and preferences with those involved in treatment and care [42–44].

As the illness situation might change towards the end of life, it is important not to understand a desire to die at home as unconditional [12]; however, advance care planning may enable discussions and nuances at an early stage and provide a realistic insight into available options. This process may also ease nurses’ sense of great responsibility.

### A ‘home place’

An interesting finding is HCPs’ pragmatic way of describing the necessity of transferring patients to a nursing home or a palliative unit in the community. After trying hard to allow a patient to die at home, HCPs might think of the transfer as implying that they had failed or given up. One reason for this pragmatism, or maybe realistic attitude, could be previous experience and knowing what is feasible with limited resources in a rural context: ‘We’re well aware of where we live’. As shown in other studies [6, 7, 45], the shortage of HCPs was the main reason for transfer in our study. A rural or remote setting often means that HCPs and family members ‘walk a tightrope’ in providing care at home for as long as possible. If the situation lasts longer than expected and planned, the care burden might be too high for district nurses, and transferring the patient to a local nursing home could ease the pressure on them [46].

In our opinion, the focus on a low rate of home deaths in Norway needs to be nuanced, and two recent Norwegian official reports on palliative care underline the importance of ‘time at home’ in the final days and weeks of life [8, 9]. This suggests that where patients live for their final 30 days may be a better quality indicator of palliative care than the actual place of death [8, 9]. Further, we propose extending ‘home’ to include ‘home place’, and thus local health care facilities. Establishing small palliative units or dedicated palliative beds in local nursing homes may be one way of strengthening primary palliative care. Palliative units or beds can provide a home-like interior including personal artefacts, which will create a link to the patient’s old home. Further, patients living in remote locations may feel isolated at home [46], and staying in a more central nursing home may fulfil their desire for social and interpersonal contact, and thus enhance their feelings of belonging and security [3].

In addition to patients’ individual needs and wishes, living far from hospitals and urban areas means that palliative care and place of death need to be adjusted to local conditions [25]. Pesut et al. [38] argue for a tailored approach to providing palliative care in rural areas, where local HCPs, patients and families could have a mutual understanding of feasibility with available resources. This approach implies that HCPs have the necessary competence and that local nurses and GPs collaborate closely [3, 34].

### Strengths and limitations

We conducted a broad, mainly purposive recruitment of 52 health professionals from primary care in rural Northern Norway, and obtained rich data from ten focus group discussions and six individual interviews. The size of the study was designed to cover local variations in the organization of primary palliative care. Three articles [25, 34, 47] have already been published from the study.

Our professional and occupational backgrounds gave valuable insight, access and credibility, and influenced the way the discussions were interpreted. Trustworthiness was assured by using an audit trail throughout the analysis process. To address credibility, we discussed our findings with colleagues with experience in palliative care. Some of the participants were known to us from professional settings, but we did not know any participants personally. The study was conducted in rural Northern Norway and in the Norwegian health care system, which may be a limitation. However, we believe that our findings may be transferable to other primary care settings in rural areas. Our data were collected in 2015-16. Since then, the health care system in Norway, as in many other countries, has been challenged by work overload and staff shortages. However, our findings are in line with earlier [39] and more recent Norwegian studies [48].

## Conclusion

Providing palliative care in rural areas involves specific professional and ethical challenges that need to be addressed. HCPs’ dual roles in small communities may place an additional burden on them, and need to be considered in the organization of care in such settings. The participants’ pragmatic attitude when transfer to a local nursing home or hospital was necessary underlines the importance of building on local knowledge and collaboration. A systematic approach using advance care planning may be one way of facilitating discussions between patients, family carers and HCPs, and thus enhancing mutual understanding of what is possible to achieve in a rural context.

### Electronic supplementary material

Below is the link to the electronic supplementary material.

Supplementary Material 1: Additional file 1: COREQ checklist

## Data Availability

The dataset analysed during the current study is not publicly available due to requirements from the Norwegian Centre for Research Data but is available from the corresponding author on reasonable request.

## Abbreviations

DN: District nurse
FGD: Focus group discussion
GP: General practitioner
HCP: Health care professional
Muni: Municipality
ON: Oncology nurse
Physio: Physiotherapist
BB: Birgit Brøndbo
