# Supplementary material for: Dying at “home” - a qualitative study of end-of-life care in rural Northern Norway from the perspective of health care professionals
Source: BMC Health Serv Res. 2023 Dec 5;23:1359. doi: 10.1186/s12913-023-10329-6 (PMC10698939; doi:10.1186/s12913-023-10329-6)
Supplement: Supplementary file 1 — Supplementary Material 1: Additional file 1: COREQ checklist [file 12913_2023_10329_MOESM1_ESM.docx]

COREQ list

Dying at “home” - a qualitative study of end-of-life care in rural Northern Norway from the perspective of health care professionals

**Bente Ervik, Tom Dønnem and May-Lill Johansen**

|  | PERSONAL CHARACTERISTICS |  |
| --- | --- | --- |
| 1 | Interviewer | Data collection, paragraph 4 |
| 2 | Credential | Method, paragraph 1 |
| 3 | Occupation | Method, paragraph 1 and data collection, paragraph 4 and 6 |
| 4 | Gender | Method, paragraph 1 |
| 5 | Experience and training | Data collection, paragraph 6 |
|  | RELATIONS |  |
| 6 | Relationship established | Strengths and limitations |
| 7 | Participants knowledge of interviewer | Strengths and limitations |
| 8 | Interviewer characteristics | Introduction and strengths and limitations |
| 9 | Methodological orientation. Theory | Introduction, theory about home |
|  | PARTICIPANTS |  |
| 10 | Sampling | Data collection, paragraph 2,4 |
| 11 | Method of approach | Data collection, paragraph 2,4 |
| 12 | Sample size | Data collection and table 1 |
| 13 | Non-participants | N/A |
|  | SETTING |  |
| 14 | Setting of data collection | Data collection, paragraph 4 |
| 15 | Presence of non-participants | N/A |
| 16 | Description of sample | Data collection, paragraph 4 and table 1 |
|  | DATA COLLECTION |  |
| 17 | Interview guide | The interview guide is provided in an Additional file in a previous paper from the same study (refm349. The interview guide was not pilot tested |
| 18 | Repeat interviews | N/A, no repeated interviews |
| 19 | Audio recording | Data collection, paragraph 6 |
| 20 | Field notes | Analysis, paragraph 1 |
| 21 | Duration | Data collection, paragraph 4,6 |
| 22 | Data saturation | N/A |
| 23 | Transcript returned | N/A |
|  | ANALYSIS AND FINDINGS |  |
| 24 | Number of data coders | Analysis, paragraph 1 |
| 25 | Description of coding tree | Available from authors on request |
| 26 | Derivation of themes | Analysis |
| 27 | Software | Analysis, no software was used |
| 28 | Participants checking | N/A |
| 29 | Quotation presented | Results section |
| 30 | Data and findings consistent | See review |
| 31 | Clarity of major themes | Result section – 4 themes |
| 32 | Clarity of minor themes | N/A – no minor themes |
|  |  |  |
